# Vomition in the Horse

**Published:** 1901-04

**Authors:** W. E. A. Wyman

**Affiliations:** Milwaukee, Wis.


### REPORTS OF CASES.

### VOMITION IN THE HORSE.

By W. E. A. Wyman, M.D.V., V.S.,
MILWAUKEE, WIS.

The valuable article on "Gastric and Intestinal Disorders o the Horse," by Veterinary Surgeon Desmond, of South Australia,[1] is brought to my memory by a case met with last week. Desmond says: "A few cases are recorded where the horse vomited and recovered," etc. My experience along that line is quite different, having seen a great number of horses suffering with acute indigestion—vomit (if such a term is at all admissible)—and recover. Chief Inspector Desmond's remarks in regard to the use of opium also permit of a controversy. Nevertheless, his paper is valuable and practical, and will be greeted with pleasure by the experienced veterinarian.

The case of vomition above referred to was as follows: The animal was a broncho belonging to a baker, and, according to the telephone message, was "running from the nose by the quart." On arrival I found a poorly-fed broncho, apparently feeling first-class. Œsophagus, pulse, respiration, temperature, and gastric and intestinal sounds normal. All at once he curved the neck, and about a quart of watery stuff mixed with bread gushed out of both nostrils. Immediately after this performance the animal started to eat some hay in front of him. I was informed that the animal had done this three or four times between the telephone call and my arrival. The floor in front of the animal was covered with the sloppy material just mentioned. Inquiry revealed the fact that about one hour before he started to vomit the animal had taken in three two-gallon pailfuls of bread soaked in water, and that he had been given that every day for about a year. Previous to this the horse had been well, nor has he missed a meal since.

Ordinarily such a report would be classed by the writer among the fake reports, but having personally encountered it and encouraged by Desmond's remarks its publication seems warranted.

[1] Journal of Comparative Medicine, etc., February, 1901, p. 71.

